# An Exploration of Factors Related to Quality of Life in Indonesian Care Workers in Home-Based Care Settings

**DOI:** 10.1097/JNR.0000000000000314

**Published:** 2019-09-20

**Authors:** Shu-Fen LO, Li-Jung CHANG, Mark HAYTER, An-Chi O YANG

**Affiliations:** 1 PhD, RN, Associate Professor, Department of Nursing, Tzu Chi University of Science and Technology, Nursing Technology Innovation Service Research Center, Taiwan, ROC; 2 MSN, RN, Assistant Professor, Department of Nursing, Tzu Chi University of Science and Technology, Taiwan, ROC; 3 PhD, RN, Professor, Nursing and Health Research, Faculty of Health Sciences, University of Hull, England, UK; 4 RN, Lecturer, Department of Nursing, Tzu Chi University, and Doctoral Program Student, School and Graduate Institute of Nursing, National Taiwan University, Taipei, Taiwan, ROC.

**Keywords:** Indonesian care workers, quality of life, stress perception, social support

## Abstract

**Background:**

Indonesians constituted 79% of foreign care workers for long-term care in Taiwan in 2015. Therefore, it is crucial to explore the effect of work stress and quality of life (QoL) on this population.

**Purpose:**

This study aims to explore stress levels, social support requirements, and perceived QoL among Indonesian care workers (ICWs) who work in home care settings.

**Methods:**

A cross-sectional design was used. Data were collected between 2014 and 2016 from a convenience sample of ICWs working in eastern Taiwan using a self-administered questionnaire that comprised the Stress Perception Scale (SPS), Social Support Scale (SSS), and World Health Organization Quality of Life-Brief scale. One hundred fifty-seven valid questionnaires were collected. The response rate was 80.51%. Data were analyzed using descriptive statistics, one-way analysis of variance, Pearson's correlation coefficient, and multiple regression analysis.

**Results:**

The results found that the average SPS of the sample was 70.50, with low QoL scores and requirements for more social support. In addition, significant and positive correlations were found between QoL and SPS, SSS, duration of patient care, and hours of care per week. Stepwise regression analysis showed that the most predictive variables for QoL were time spent caring, preservice training, psychological stress, and emotional support, which together accounted for 60.9% of the total variance.

**Conclusions/Implications for Practice:**

This study facilitated understanding of the stress on ICWs and the impact of social support on their QoL. The findings suggest that new immigrants in Taiwan should be introduced to foreign care workers or nursing attendants. Moreover, long-term-care-related teachers should work with home care institutions or agencies to develop a teaching model for innovative care skills to relieve the perceived stress of ICWs.

## Introduction

The world population in 2017 comprised an estimated 962 million older adults (≥ 60 years old), a population that is growing faster than all other age groups. The number of older adults worldwide is expected to more than double by 2050 and more than triple by 2100 (United Nations, 2018). This will increase workforce demands and the need for services provided by care workers. As the care burden increases, the number of foreign care workers (FCWs) increases, particularly in home care settings where FCWs are hired to alleviate the long-term-care (LTC) loading on family members (Workforce Development Agency, Ministry of Labor [MOL], Taiwan, ROC, 2017a; Wu, Lee, Lin, & Wai, 2011). FCWs are a vital and indispensable element of the workforce in current LTC systems. Since 1992, the Taiwanese government has allowed FCWs to care for people with physical or mental disorders or disabilities to meet the domestic labor shortage and the need for LTC workers (Ministry of Health and Welfare, Taiwan, ROC, 2017). However, in Taiwan, only 6.16% of FCWs work in LTC institutions (Workforce Development Agency, MOL, Taiwan, ROC, 2017b). In 2017, 76% of FCWs were Indonesians aged between 25 and 44 years (Workforce Development Agency, MOL, Taiwan, 2017b). However, many FCWs receive only short-term care training before coming to Taiwan. Thus, their care skills are not sophisticated enough to manage serious illnesses and disabilities, especially in home care settings (Chen, Liu, Li, & Kao, 2012). Most are able to speak only very limited Mandarin or Taiwanese (Huang & Yang, 2011). Inexperience, culture shock, living habits, and language problems result in conflicts between FCWs and their employers (Chen et al., 2012), which affects the quality of care provided by FCWs and their ability to adjust to their work responsibilities (Huang & Yang, 2011). This conflict of culture and language as well as the challenges of care contribute to the stress perceived by FCWs. A previous study reported that stress has a negative impact on FCWs' quality of life (QoL) and affects the quality of care they provide to patients (Chen et al., 2012; Huang & Yang, 2011). Occupational stress has harmful effects on individuals in terms of health, physical and psychological well-being, and job satisfaction, which may affect the quality of care provided to patients (Chen et al., 2012). Some studies have found that reducing occupational stress and strengthening social support and rational coping may improve QoL among FCWs (Chen et al., 2012; Wu et al., 2011). Previous studies have focused on experiences of FCWs in hospitals or LTC institutions. However, studies related to Indonesian care workers (ICWs) working in home care settings are limited. The primary purpose of this study was to examine the level of occupational stress, social support, and QoL among ICWs.

### Foreign Care Workers' Stress

Lazarus (1991) defined stress as a relationship between individuals and environment. It is a function of the degree of person–environment fit (Lazarus & Folkman, 1984). Stress is a dynamic process that occurs when an individual appraises situational demands as exceeding available resources (Lazarus, 2006). Stress may be caused by either internal or external sources. The perception of stress is conceptually distinct from the amount of stress experienced by an individual and may affect health (Keller et al., 2012). Potential stressors include work stress, work overload, and limitations on resources (Admi & Moshe-Eilon, 2010). Higher levels of stress may have a negative impact on working ability. Extended periods of work stress may have psychological or physical effects, cause behavioral changes, deteriorate quality of work, increase the risk of accidents, and even endanger patient safety (Hung & Chiou, 2013).

For FCWs, stressors include workload, care abilities, and physical load. FCWs are often required to care for patients with dementia or stroke for 24 hours, particularly for patients with multiple physical or mental disorders who often need help to change position or use the toilet during the night (Huang & Lin, 2013; Yang, Wang, Wang, & Wang, 2013). FCWs average 10.2 hours/day at work (Workforce Development Agency, MOL, Taiwan, ROC, 2017b). Studies indicate that FCWs who take care of patients with functional deficits, have less than 6 months of experience working in Taiwan, or are not proficient in Chinese are more likely to have a higher level of stress perception (Wu et al., 2011). With respect to care ability, FCWs receive only 90 hours of training before coming to Taiwan (Fu, 2018). Moreover, their training addresses only basic communication skills, occupational safety, health and inspection, Taiwan work policy, and housekeeping issues (Ministry of Labor, Taiwan, ROC, 2018). Most FCWs do not receive a care training program, but FCWs' care experiences affect patient safety and quality of care. Nearly 60% of FCWs perform noninvasive techniques such as measuring blood pressure and body temperature. They also perform invasive care such as feeding medicines or milk using a nasogastric tube and sputum suction (Yang et al., 2013). These techniques are challenging for FCWs and may endanger the safety of patients if performed incorrectly (Chen, Lu, Chen, & Li, 2014).

With respect to physical load, nearly 70% of FCWs sleep less than 8 hours a day (Huang & Lin, 2013). When FCWs have insufficient time for rest, stress increases, which subsequently affects quality of care. Consequently, care accidents such as repeat hospitalizations, unexplained infections, and pressure injuries may increase (Kao, Chen, & Yang, 2013). The error rates for nasogastric tube care and trachea care are 58% and 57%, respectively (Chen et al., 2014). The stress level of FCWs affects not only perceptions of stress but also quality of care. Stress is a major factor contributing to poor quality of care and QoL in FCWs.

### Social Support Needs for Foreign Care Workers

When FCWs work in an unfamiliar environment without sufficient care experience, they experience poor life adjustment and perceive greater stress. Receiving appropriate support and assistance may improve coping strategies (Koinis et al., 2015). Social support needs include emotional support in terms of self-esteem, love, compassion, listening, and comfort; instrumental support such as money, material assistance, and action intervention; and informative social support such as advice, recommendations, guidelines, and other helpful messages. A study revealed that FCWs are prone to mental health problems or substance abuse if they feel disrespected or experience poor social support (Huang & Yang, 2011). Hu, Yeh, and Wang (2009) surveyed 26 discrete social support needs of FCWs and found the demand for psychosocial support to be the most prevalent. More than 50% of FCWs required financial support, family support, stress adjustment, and emotional support. Learning caring and communication skills from their employers through support groups holds the potential to significantly increase the social support received by FCWs, resulting in enhanced quality of care.

### Quality of Life of Foreign Care Workers

In 1948, the World Health Organization (WHO) defined the concept of “quality of life” as physical, psychological, social, and spiritual comfort. Thus, QoL may be defined in terms of subjective perceptions determined by culture and value systems, which includes physical function, mental status, independence, social relationships, personal beliefs, and environment. Different individuals have different interpretations of QoL (Yao, 2002). Chen et al. (2012) surveyed 300 FCWs in a medical center and nursing home in northern Taiwan. They found that younger FCWs had poor QoL and had been trained for a short period of time only, and some had not received the care training program at all. Critical predictors of QoL were available time for rest and sleep, extra bonuses or rewards, and having children. Studies indicate that job stress, social support, and self-perceived QoL dramatically affect the quality of care provided by FCWs. However, research on the stress factors affecting ICWs in home environments with respect to social support and QoL are limited. Thus, the impact of care stress and QoL in this population must be investigated.

In particular, this study was intended to answer the following research questions: (1) What are the major sources of occupational stress among ICWs? (2) What are the levels of occupational stress, social support, and QoL among ICWs? (3) What are the relationships between demographic characteristics and occupational stress, social support, and QoL in ICWs? and (4) What are the variables that best predict QoL in ICWs?

**Figure 1.**
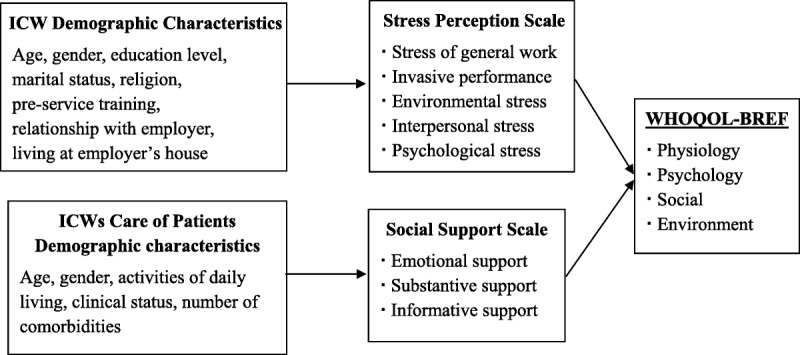
The conceptual framework of quality of life and its related factors among Indonesian care workers (ICWs). WHOQOL-BREF = World Health Organization Quality of Life-Brief Scale.

The conceptual framework was developed for this study by integrating information from the studies in the literature review (Figure 1).

## Methods

### Study Participants and Setting

This study employed a cross-sectional design. The ICWs were recruited from five nursing home agencies and three foreign worker agencies in eastern Taiwan between August 1, 2014, and July 31, 2016. Inclusion criteria for the ICWs were (1) aged 20 years or older; (2) able to read the Indonesian version of the questionnaire; (3) involved in the informal care of patients (e.g., helping feed and wash patients) in home care settings; and (4) willing to participate in the study. This study was approved by the Ethics Committee of the Hualien Tzu Chi Hospital (IRB No. 104–28-B); 195 participants were recruited, and 157 completed the questionnaires, a response rate of 80.5%.

### Instruments

Demographic characteristics of the ICWs, including age, gender, and education level, and the marital status and demographic and disease characteristics of their patients were collected. As discussed in the following paragraphs, three instruments were used in this study.

#### Stress perception scale

Participant stress levels were assessed using the 29-item Stress Perception Scale (SPS; Chen et al., 2012, 2014; Wu et al., 2011), which was designed to measure stress experiences during the previous 1-month period. Total possible SPS scores range from 29 to 145, with higher scores associated with greater stress. After factor analysis, five factors were extracted: “stress of general work” (9 items), “invasive performance” (7 items), “environmental stress” (3 items), “interpersonal stress” (5 items), and “psychological stress” (5 items), which explained 77.22% of the variance. The Cronbach’s α was .94.

#### Social support scale

The Social Support Scale (SSS) was used to measure the social support requirements of the ICWs while they provided care to patients (Lo, Hwang, Yau, & Liu, 2001; Wang, Yang, Chen, & Wang, 2013). The SSS comprises 17 items. The range of possible scores is from 17 to 68, with higher scores indicating higher levels of perceived social support. After factor analysis, three factors were extracted: “emotional support” (8 items), “substantive support” (5 items), and “informative support” (4 items), which explained 74.22% of the variance. The Cronbach’s α was .91.

#### WHO Quality of Life-Brief Taiwan version

The WHO Quality of Life-Brief (WHOQOL-BREF) was used to measure the QoL of the ICWs. This tool comprises 28 items, including physiological, psychological, social, and environmental factors, as well as two Taiwanese localization issues (Yao, 2002). The range of possible WHOQOL-BREF scores is from 16 to 80, with higher scores indicating better QoL. After factor analysis, the five factors explained 55.89% of the variance. The Cronbach's α was .87.

### Statistical Analyses

Data were analyzed using IBM SPSS Statistics Version 19.0 (IBM, Inc., Armonk, NY, USA). Descriptive statistics were used to assess the participants and the general characteristics of their patients. One-way analysis of variance was used to verify the differences in QoL according to the general characteristics. Post hoc analyses were performed using Scheffe's test. The Pearson correlation coefficient was used to analyze the correlations among the SPS, SSS, and QoL of the participants. Finally, multiple regression analysis was used to identify the factors that influenced the QoL of the participants, the alpha level was set to *p* < .05.

## Results

### Sample Characteristics

The age of the study population ranged from 23 to 49 years (mean: 35.46 ± 6.54 years). Among the participants, 80.25% were married and Muslim. The most common educational level was junior high (55.41%), and 79.62% lived at their employers' house. The average experience with patient care was 2.74 ± 1.98 years. The average time spent on caring was 15.97 hours/day, and the average duration of sleep was 6.30 hours/day. In addition, 84.72% had not received nursing training before coming to Taiwan. Their roles varied and included caring for older adults (57.5%), cleaning the house (65.0%), and preparing meals (60%; Table 1). The average age of the patients was 78.9 years, most were male (52.22%), and 44.59% required assistance with activities of daily living (Table 2).

**TABLE 1.**
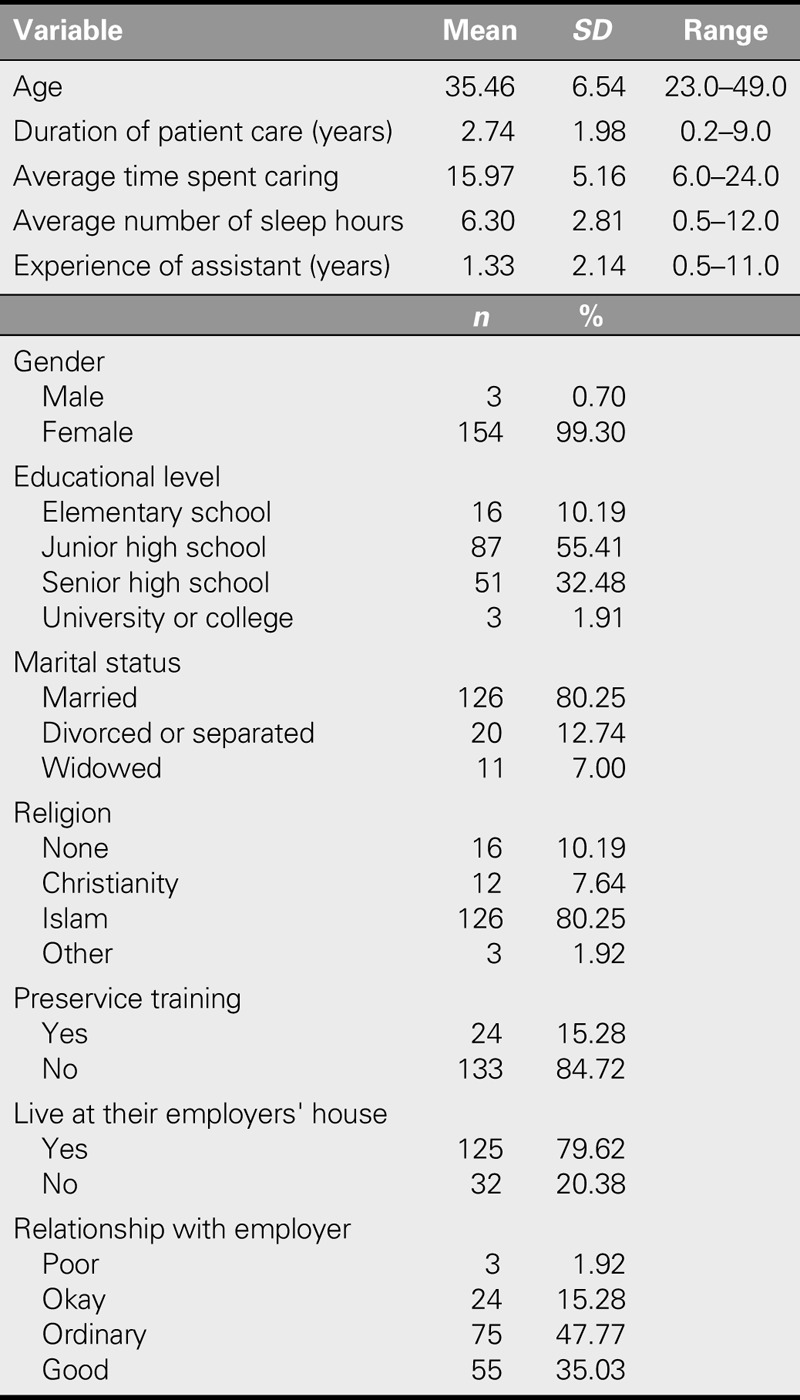
Demographic Characteristics of Indonesian Care Workers (*N* = 157)

**TABLE 2.**
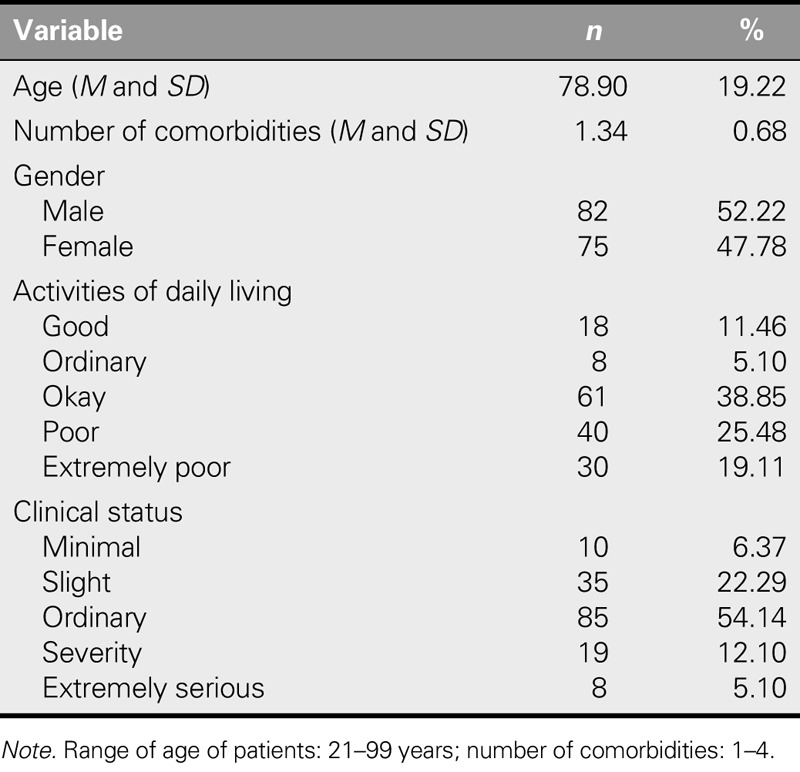
Demographic and Disease-Related Characteristics of Indonesian Care Workers of Patients (*N* = 157)

### Descriptive Analysis

Table 3 presents a descriptive analysis of the SPS. The SPS scores ranged from 27 to 111, with an average stress pressure score of 70.50 (*SD* = 22.99). The stress intensity item with the highest score was psychological stress. The lowest score was for the invasive performance domain. Table 3 shows the participants' subjective perceptions of their social support needs. The SSS ranged from 17 to 68. The mean score was 50.98 (*SD* = 10.18), indicating that the degree of social support requirement was moderate, but the difference was quite large. The most important need for ICWs was emotional support, with the lowest scores in the substantive social support domain. Table 3 also presents a descriptive analysis of the WHOQOL-BREF scores, the total scores for which ranged from 41.76 to 66.70, with a mean score of 56.87 (*SD* = 5.19). The average scores for each domain were divided by 4 to calculate the scores in a range from 4 to 20. The highest score was for the environmental domain (14.67 ± 1.93), and the lowest score was for the physiological domain (12.96 ± 1.60).

**TABLE 3.**
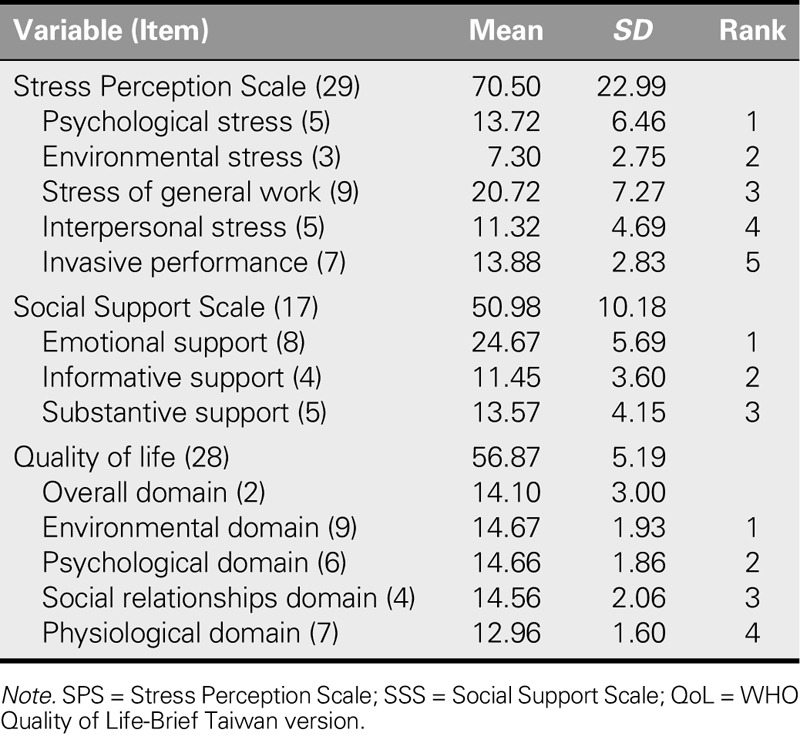
The Mean and Standard Deviation of the SPS, SSS, QoL, and Each Domain (*N* = 157)

### Relationships Between Independent Variables and QoL

The factors influencing QoL were analyzed using one-way analysis of variance. Scheffe's post hoc test revealed that the QoL of participants who had received orientation training before coming to Taiwan was higher (*F* = 6.76, *p* = .013). The relationships between QoL and demographic variables, SPS, and SSS were analyzed, with results showing that stress perception, social support, duration of patient care, and hours of care per week were all significantly correlated with QoL. The correlation coefficients were .45, .28, .46, and .86 (*p* < .05), respectively, indicating that higher levels of pressure were associated with lower levels of social support received. In addition, longer lengths of care and more hours of care per week were both associated with poorer self-perceived QoL (Table 4).

**TABLE 4.**
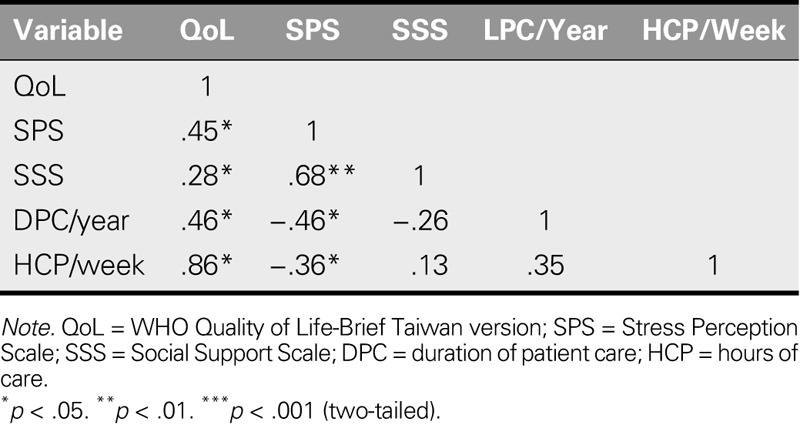
Correlation Between QoL and Variables (*N* = 157)

### Predictors of Quality of Life

Multiple regression analyses were conducted to analyze all of the independent variables based on the significance of the results from the Pearson's correlation analyses. The discontinuous variables were converted into dummy variables before the analysis. The results revealed that average time spent caring (*F* = 5.30, *p* = .032), not receiving complete training in patient care before coming to Taiwan (*F* = 6.76, *p* = .013), psychological stress (*F* = 6.25, *p* = .017), and emotional support (*F* = 4.81, *p* = .035) were all significant variables determining QoL, accounting for 60.9% of the total variance (Table 5). The duration spent caring each day and not receiving complete training before coming to Taiwan strongly predicted QoL in the participants.

**TABLE 5.**
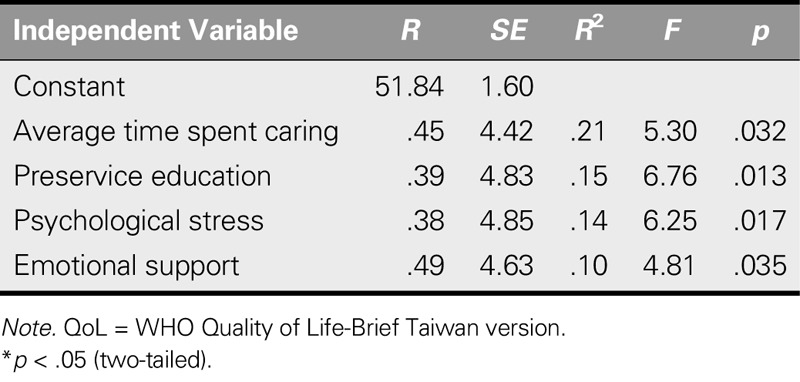
Regression Analysis Variables Predicting QoL Total Scores (*N* = 157)

## Discussion

Previous studies have revealed low levels of stress among FCWs (Chen et al., 2012). However, this study found that the ICW participants were exposed to moderate levels of stress and that level of stress varied significantly among individual participants. Possible reasons for this variance include differences in previous working experience, the average number of hours of care provided per day, and the age of their patients. Average nursing experience was 1.33 years, and the average age of patients was 78.90 years. In addition, 44.57% of the patients had poor self-care ability. Most of the participants (84.72%) had no preservice training. According to the policy of the Ministry of Labor, Taiwan, ROC (2018), the standard 90-hour pretraining program includes only language, occupational safety, health and inspection, Taiwan work policy, and housekeeping training. However, ICWs are otherwise required to possess nursing skills such as nasogastric tube care and trachea care, which may be sources of considerable stress. The daily duration of work was very long, with 84.4% of FCWs working in excess of 10 hours/day (Wang et al., 2013; Wu et al., 2011).

### Stress Perception of ICWs

Scores were high at all levels of stress perception; however, psychological stress scores were the highest. The reasons for the latter were likely associated with autonomy of care, as the most significant psychological stress events identified in this study were “having no right to decide care job by themselves” and “facing harassment from employers.” These two factors are closely linked to the fact that ICWs work and live in the same home care setting. As ICWs have little input into care-related decisions, work overload is likely to increase stress and thus affect the quality of care provided (Jetha, Kernan, Kurowski, & Pro-Care Research, 2017). The findings of this study suggest that employers should give ICWs appropriate decision-making authority to make care-related decisions based on their knowledge of care and length of stay in Taiwan. Moreover, employers should consider them as family members and address their concerns (Kang & Lee, 2010). The perception among FCWs of being harassed is also important (Hanson, Perrin, Moss, Laharnar, & Glass, 2015). FCWs should be aware of their labor rights and know how to seek help and support through channels such as the FCW 24-hour counseling and protection hotline.

### Social Support Needs of ICWs

In this study, the social support needs of the participants were rated in the middle to upper range (*M* = 50.98), with considerable difference among individuals. The ICWs in our study seemed to be socially isolated. This feeling of isolation may be because of language barriers, which complicates seeking help from others (Kang & Lee, 2010). The results regarding social support in this study were congruent with those of previous studies (Chen et al., 2012). FCWs feel socially isolated because of the care needs of older patients, which may occur 24 hours/day without support (Chen, 2011). We found that 55.41% of the subjects reported junior high as their highest level of education and that only 40% could speak Mandarin. These individuals require considerably more support, especially emotional support, in the working environment. Furthermore, the participants required spiritual counseling and assistance.

### QoL of ICWs

The QoL scores of the ICWs were moderate (mean: 56.87 ± 5.19), which is inconsistent with Chen et al. (2012), who reported a mean overall QoL score of 68 (*SD* = 11.34), with the highest score in the social domain and the lowest score in the environmental domain. This may be because of differences in average daily working hours. In this study, the average was 15.97 hours/day, much higher than the average reported by Chang Chien, Lee, Sun, Chou, and Yang (2012). Previous studies indicate that working hours, educational level, age, number of days off, and length of working experience each affect the QoL of FCWs (Chang Chien et al., 2012). In this study, the highest QoL subscale was the environmental domain and the lowest score was for the physiological domain. Previous studies indicate that FCWs often work for more than 12 hours/day, which requires considerable physical strength and spiritual stamina. This work pressure had an adverse impact on the time available for mental/physical rest and worsened the physical and mental health of ICWs. This poor work situation affected their QoL (Chang Chien et al., 2012; Huang & Lin, 2013).

In addition, this study found that the top three QoL items for the participants were “feeling my life was meaningful,” “satisfaction with my current life,” and “satisfaction with my environmental health.” The lowest three items were “having frequent, negative feelings,” “having little time to engage in leisure activities,” and “reliance upon medical help to cope with daily life.” The results of this study differed from those of Chen et al. (2012), who reported that the highest scored item for ICWs was “having frequent, negative feelings” and the lowest scored items were “having little time to engage in leisure activities” and “reliance on medical help to cope with daily life.” Most ICWs worked to improve their lives, and they earned a substantial amount working overseas to help them achieve this goal. This may be the reason they felt their lives were meaningful and satisfactory. The negative feelings and low leisure activity may be because of frustrations related to their jobs and to working overtime. Previous studies have found that, when FCWs are overburdened and unable to take leave, negative feelings of inability are easily induced (Huang & Lin, 2013).

### Predictors of Participant QoL

FCWs play a significant role in providing direct care to elderly patients in long-term home-based caregiving. The job stress, social support, and QoL of FCWs are factors crucial to the provision of quality patient care. This study found that the key determinants for high QoL among ICWs were social support, preservice training, and ability to cope with stress. Hours of care per week and average time spent in caregiving were also important predictors. These results were consistent with those of other studies (Chang Chien et al., 2012; Chen et al., 2012), which revealed that shorter working hours, more adequate rest, and appropriate vacation periods reduce stress and enhance physical recovery (Ha, Chong, Choo, Tam, & Yap, 2018). Working overtime and having little rest cause stress and affect the quality of care provided by FCWs (Chang Chien et al., 2012; Huang & Hsieh, 2010; Li, Ho, & Che, 2015). Chang Chien et al. (2012) noted that FCWs should have reasonable working hours and adequate rest to improve their care quality and QoL. The 2017 Foreign Nursing Workers Respite Service Program in Taiwan was intended to address these problems and help enhance the QoL of FCWs.

Appropriate training programs are essential to equip FCWs with the skills necessary to provide competent home-based patient care. Relevant training includes disease-specific caregiving skills, language training opportunities, information about available support groups, recognition of the early signs of depression, and stress management (Wu et al., 2011; Yeoh & Huang, 2010). Huang and Hsieh suggested that FCWs should undergo training courses to strengthen their caregiving skills and knowledge before beginning work. However, FCW in-service education faces several barriers, including lack of approval from Taiwanese employers, time limitations, and employer unwillingness to pay for the costs of this education (Li et al., 2015). Therefore, the use of intelligent technology (e.g., apps and QR codes) with Indonesian dubbing is proposed to help ICWs learn care skills and knowledge.

FCWs alleviate the physical, mental, and behavioral impairments of ill older adults in home care settings (Østbye, Malhotra, Malhotra, Arambepola, & Chan, 2013). However, the results of this study indicate that the psychological stress experienced by ICWs has a negative impact on their QoL. Strong evidence from several studies has shown that long work hours, multiple roles (e.g., household chores, childcare, and caregiving for the older adults), work overload, client disability care, restrictions on mobility and communication, and conflict between patients and their families affect FCWs and eventually become sources of psychological stress and burden (Ho, Chiang, Leung, & Ku, 2018; Wu et al., 2011). Taiwan's policymakers must devise standardized occupational strategies to enhance the working conditions of FCWs, such as rest days, proper accommodation, and safe working conditions.

Social support is especially crucial in mediating stress and improving QoL for FCWs (Chang Chien et al., 2012; Tam, Koh, Legido-Quigley, Ha, & Yap, 2017). Individual well-being, achieved through socioemotional support of personal capacities, may mediate stress (Østbye et al., 2013). A study in Singapore found that FCWs coped better with caring for an ailing elderly person when they received support from that person's family (Ha et al., 2018). The quality of care that FCWs provide may be expected to improve when employer–employee relationships are relatively equal (Yeoh & Huang, 2010). Ideal care worker–care receiver relationships rely on active but noninterfering participation by the families of both the care worker and the care receiver. The development of kin-like relationships with care receivers may enhance FCWs' emotional support and job satisfaction, alleviate burnout, and improve QoL (Chang Chien et al., 2012; Ho et al., 2018).

### Limitations

This study has several limitations. First, data were collected in eastern Taiwan. Thus, these results may differ from those from FCWs of different cultural or religious backgrounds. Second, during this study, the Taiwan government's Long-Term Care 2.0 policy promoted FCW respite services and home-based supplementary training programs. This study could not identify the areas where this pilot project was being conducted. Future studies should be conducted to compare the provision of care in areas where the Long-Term Care 2.0 policy is and is not in effect. The cross-sectional nature of this study could not provide insight into the stressors, social support requirements, and QoL in the ongoing dynamic processes of FCWs. Therefore, longitudinal studies are required to further understand the support and assistance requirements of FCWs.

### Conclusions

This study showed that the participants experienced moderate to high degrees of stress at work and perceived a low level of social support. The need for “emotional social support” was high, and participants needed to “be encouraged when frustrated,” to “have someone to talk to when they encounter problems,” and to have contacts with “someone who understands the situation they experienced.” They often experienced negative feelings and relied on medical help to cope with daily life and did not have sufficient time to engage in leisure activities. In addition, receiving more adequate preservice training and working for reasonable amount of time per day were shown to have a positive effect on QoL. In Singapore, the Foreign Domestic Worker Association was established to help FCWs (Choon, 2017). We suggest establishing a similar association to provide skills training through affordable training programs and social support to FCWs.

At present, Chinese and Indonesian versions of care manuals are available online for healthcare workers to download. However, the care skills presented in the manuals are very complex, and FCWs who lack formal training require longer time to learn. Because most ICWs have smartphones, applications may be used to link training and care delivery resources with QR codes to make audio and video care-skill and other related resources available for download. Recent legislation has been enacted to promote the planning of respite programs for foreign nursing aides (Ministry of Health and Welfare, Taiwan, ROC, 2017). After the implementation of these programs, studies should address the effectiveness of this policy as a means of reducing work-related stress in FCWs.

Because most ICWs are Muslim, they cannot eat pork or food that has been used in worship ceremonies. However, most Taiwanese do eat meat and consume food used in traditional worship services. As the lifestyle requirements of ICWs differ from the mainstream in Taiwan, their beliefs and lifestyles should be understood and respected. Employers should consider ICWs as family members as a strategy for securing high-quality care. Furthermore, the Taiwan government should facilitate the provision of spiritual counseling and assistance to reduce the stress of FCWs through their LTC policy. In recent years, the Taiwanese government has emphasized cooperation between industry and educators. The impact on LTC-related education should be explored in the future to improve care standards, teach critical care skills, and empower FCWs. The results of this study provide information for healthcare professionals and policymakers involved in improving home-based healthcare in Taiwan as well as those involved in the welfare and training of FCWs.
